# A qualitative systematic review of umbilical cord care practices in Nigeria

**DOI:** 10.1186/s12887-025-05387-0

**Published:** 2025-01-15

**Authors:** Precious Ebube Anyakorah, Florence Chinelo Aguna, David Chinaecherem Innocent, Anthony Chinonso Uwandu-Uzoma, Uzochukwu Godswill Ekeleme, Chidera Chisom Obasi, Stanley Chinedu Eneh, Chidinma Peace Ahunam, Ihuoma Chimdimma Dike, Vivian Chidimma Maduekwe, Oluwaseunayo Deborah Ayando, Chinazaekpere Oguguo Duruji, Rejoicing Chijindum Innocent, Princess Chidiebube Uwaezuoke, Oluwafunmilayo Opeyemi Adenuga, Chiagoziem Ogazirilem Emerole

**Affiliations:** 1 Department of Public Health, Federal University of Technology Owerri, Owerri, Imo State Nigeria; 2 Department of Dental Technology, Federal University of Technology Owerri, Owerri, Imo State Nigeria; 3 Department of Nursing & Healthcare Leadership, Faculty of Health Studies, University of Bradford, West Yorkshire, UK; 4 Department of Community Health, Faculty of Clinical Sciences, Obafemi Awolowo University, Ile-Ife, Osun State Nigeria; 5 Department of Public Health, Kwara State University, Malete, Kwara State Nigeria; 6 Department of Health Promotions, Community Healthcare Network Hospital, Isiala Mbano, Imo state, Nigeria; 7 Department of Pharmacy, Enugu State University of Science and Technology, Enugu State, Nigeria; 8 Department of Pharmacy, University of Nigeria Teaching Hospital, Ituku-Ozalla, Enugu State, Nigeria

**Keywords:** Umbilical cord, Cultural practices, Healthcare infrastructure, Socioeconomic factors, Nigeria

## Abstract

**Background:**

Umbilical cord care is an important aspect of newborn health, and different practices exist around the world, often influenced by cultural, healthcare infrastructure, and socioeconomic factors. The objective of this systematic review is to synthesize current literature on umbilical cord care practices in Nigeria, with an emphasis on the impact of cultural beliefs, healthcare infrastructure, and socioeconomic factors.

**Methods:**

A comprehensive search for literature was performed across PubMED, MEDLINE and Google scholar for studies published between 2010 and 2023. The preferred reporting items for systematic reviews and meta-analysis (PRISMA) guidelines was followed for the execution of this study. Eligibility criteria included only English studies investigating umbilical cord care practices in Nigeria, with outcomes connected to cultural, healthcare, or socioeconomic factors. Critical Appraisal Skills Programme (CASP) checklist was used to critically appraise the quality and rigor of selected studies. Due to the heterogeneity of the studies (qualitative and quantitative), qualitative narrative synthesis was used to synthesize the studies in a textual format for comprehensive understanding.

**Results:**

A total of 11 included studies were found out of 1532 studies. The findings reveal a range of cord care practices, emphasizing the use of various methods such as methylated spirit, hot compresses, and indigenous substances. Cultural beliefs, a lack of healthcare infrastructure, and socioeconomic circumstances all have a big impact on cord care decisions. Disparities in knowledge and adherence to evidence-based procedures are noticeable, particularly in the use of chlorhexidine gel. Infections continue to be a problem, highlighting the significance of appropriate therapies.

**Conclusion:**

This systematic review offers a comprehensive perspective of Nigerian umbilical cord care practices, emphasizing the importance of culturally responsive educational interventions, enhanced healthcare infrastructure, and targeted legislative measures. Despite its limitations, the study is an important resource for guiding future research, policy creation, and interventions to improve maternal and newborn health outcomes in Nigeria.

## Introduction

In the context of neonatal health, proper umbilical cord care and management are critical to ensuring neonates’ well-being [1, 2]. The umbilical cord serves as a lifeline for infants, linking them to vital nutrients and oxygen during gestation [2]. The first 28 days of life, known as the neonatal period, are a vital period in which babies become particularly vulnerable to several health risks [3]. However, once the infant is born, good umbilical cord care is critical to preventing infections and ensuring a smooth transition to extrauterine life [3]. According to Coffey and Brown [4], germs can enter the newly cut umbilical cord and cause newborn sepsis and mortality. Essential newborn care, encompassing practices like umbilical cord care, is shaped by cultural norms [1]. These practices, ranging from elaborate rituals to minimalist approaches, reflect diverse community values [1]. Cord infections are becoming more common and widespread in developing countries as a result of poor cord care practices [5, 6]. In most developing countries, the prevalence of newborn cord infection ranges from 3 to 5% [4]. Proper umbilical cord care is crucial for minimising the prevalence of infections and related issues, which eventually contributes to lower infant death rates [1].

Cultural influences significantly shape practices and attitudes around umbilical cord care [7, 8]. Diverse cultural perspectives dictate various methods for managing the newborn’s umbilical cord stump during the postnatal period [9, 10]. These practices often stem from deep-rooted traditions and cultural norms that reflect community values and beliefs [11]. For instance, some cultures emphasize elaborate rituals involving specific herbs, oils, or ceremonies to promote the newborn’s health and smooth transition to infancy [8]. Conversely, other cultures adopt a minimalist approach, allowing the natural healing process to occur with minimal intervention [7]. This variation underscores the broad spectrum of umbilical cord care practices across different communities.

Nigeria has a diversified socioeconomic landscape, with major variations in income, education, and healthcare infrastructure [11]. Families with limited financial resources may face barriers to receiving appropriate healthcare treatments, resulting in variances in understanding and adherence to suggested cord care procedures [12]. However, disparities in wealth, education, and geographical location can influence parents’ decisions about neonatal care, particularly umbilical cord care practices [13, 14]. Education levels and awareness are important factors to consider, since those with greater education may be more likely to acquire and interpret healthcare information [15]. Furthermore, variations in urban and rural healthcare infrastructure can affect the availability of qualified clinicians and the necessary resources for adequate umbilical cord care [12].

Nigeria’s heterogeneous healthcare system, characterised by disparities in resources, accessibility, and quality of care, has a substantial impact on the adoption of evidence-based practices in neonatal health [16]. Understanding the state of healthcare facilities, ranging from well-equipped metropolis hospitals to under-resourced rural clinics, is critical for measuring compliance with suggested umbilical cord care recommendations [17]. In urban regions with more modern medical facilities, there may be a higher possibility of adherence to evidence-based procedures, such as the use of sterile tools and antiseptic chemicals [10, 14]. However, in isolated locations with limited access to healthcare resources, traditional or home-based practices may be more common due to a lack of medical direction [15]. The purpose of this systematic review is to offer evidence on different umbilical cord care practices in Nigeria and identify evidence-based practices that can provide effective umbilical cord care in Nigeria.

### Rationale/ justification

Every year, around 4 million newborn deaths occur worldwide, with approximately 99% of these mortalities occurring in low and middle-income countries (LMIC), notably Sub-Saharan Africa (SSA) [17, 18]. Sepsis causes around three-quarters of all neonatal deaths [18], with infections of the umbilical cord responsible for a considerable rate of infections in Sub-Saharan Africa [3, 19, 20]. Numerous hospital-based studies in Nigeria found incidences of umbilical cord infections responsible for between 10% and 19% of neonatal hospitalisations and up to 30–49% of neonatal mortality [21]. Recognising umbilical infections as a major cause of infant fatalities in Nigeria, practice recommendations on handling umbilical cords have been established [22], necessitating the study. Following an exhaustive search for literature to identify gaps in knowledge, other systematic reviews conducted by numerous researchers were discovered, including: a systematic review of umbilical cord clamping practices globally [23]; a systematic review of umbilical cord care among carers in Africa [24]; a systematic review of umbilical cord-care practices in low- and middle-income countries [4]; and a systematic review of neonatal care practices in Sub-Saharan Africa [25]. However, there has been no available evidence on the umbilical cord care practices in Nigeria; therefore, this study is required to address the knowledge gap.

This study will reveal the various practices in different parts of Nigeria for the care of the umbilical cord. This evaluation of umbilical cord care in Nigeria is further justified by the country’s need for evidence-based practices to improve infant health outcomes. The review will explore the umbilical cord care practices in Nigeria and will be useful to traditional birth attendants, midwives, other healthcare professionals, government bodies, and non-governmental bodies, thereby reducing neonatal mortality and morbidity in the country.

## Methods

### Eligibility criteria

The PEO framework (Population, Exposure, and Outcome) was utilised to develop the Eligibility Criteria of this study on Umbilical cord care practices in Nigeria [26]. Table 1 below depicts findings for the eligibility criteria of the study.

**Table 1:** Eligibility criteria

Items	Inclusion criteria	Exclusion criteria
Population (P)	- Neonates (newborn babies) in Nigeria - Mothers and caregivers in Nigeria	- Neonates outside of Nigeria - Neonates with severe medical conditions requiring specialized care
Exposure (E)	- Studies on various umbilical cord care practices including but not limited to: Dry cord care, Application of antiseptics or traditional remedies, Timing of cord separation	- Studies not focused on umbilical cord care practices in the context of newborn care in Nigeria,
Outcome (O)	- Information related to the effectiveness, safety, and cultural considerations of umbilical cord care	- Studies with no relevant outcomes or findings related to the context of newborn care in Nigeria
Design	Cross-sectional Studies Randomized Controlled Trials (RCTs), Prospective Cohort Studies, Comparative study design, Qualitative Studies, Case Reports and Case Series	Animal Studies, Studies Lacking Relevant Outcomes
Study duration	- Studies published from 2010 till date	- Studies published earlier than 2010
Language	English articles	Non-English Articles
Publication type	Original peer reviewed Articles	- Preprints, short communications, and consensus reports. - Non-original articles (e.g., review articles, meta-analyses).

### Search strategy

The study’s search method was developed using the Medical Subject Headings [MESH], synonyms and free text terms to standardize, improve precision, and ensure complete retrieval of important literatures [27]. A search was conducted on the following databases by PEA, F.

CA, SCE, COD and OOA.

- PubMED.
- MEDLINE.
- Google scholar.

A search was performed on the reference list of some potential studies. Boolean operators (OR, AND, NOT), brackets, truncations, proximities, and wildcards were used to combine or exclude search terms allowing for a more accurate and thorough retrieval of pertinent data from databases by defining connections between search terms and improving the outcome of searches. Table 2 shows the comprehensive search strategy for the study. Also, hand searching was performed by PEA ICD, VCM, and PCU.

**Table 2:** Search strategy

S/*N*	Primary Keyword	Boolean Operators (Joining Type)	MESH Terms/Synonyms/Free Text terms	Databases	Hits
1	Umbilical cord	“OR”, “(….)”	((“Umbilical Cord”) OR (“Umbilical Cord Diseases”) OR (“Umbilical Cord Compression”) OR (“Umbilical Cord Prolapse”) OR (“Umbilical Cord Clamping”) OR (“Umbilical Cord Blood Stem Cells”) OR (“Umbilical Veins”) OR (“Umbilical Arteries”) OR (“Umbilical Hernia”) OR (“Umbilicus”))	PubMED MEDLINE Google scholar	446,007
2	Care	“OR”, “(….)”	((“Patient Care”) OR (“Primary Health Care”) OR (“Health Care Quality”) OR (“Patient-Centered Care”) OR (“Palliative Care”) OR (“Critical Care”) OR (“Self Care”) OR (“Continuity of Patient Care”) OR (“Child Care”) OR (“Maternal Health Services”) OR (“Home Care Services”) OR (“Long-Term Care Concern”) OR (“Attention”) (“Tending”) OR (“Nursing”))	PubMED MEDLINE Google scholar	385,054
3	Practices	“OR”, “(….)”	((“Routines”) OR (“Habits”) OR (“Customs”) OR (“Procedures”) (“Traditions”) OR (“Techniques”) OR (“Behaviors”) OR (“Patterns”))	PubMED Medline Google scholar	6,724,014
4	Nigeria	“OR”, “(….)”	(Nigeria)	PubMED Medline Google Scholar	
5	(1) AND (2) AND (3) AND (4)			Medline Google scholar	1,532 (11)

### Study selection

**Fig. 1:**
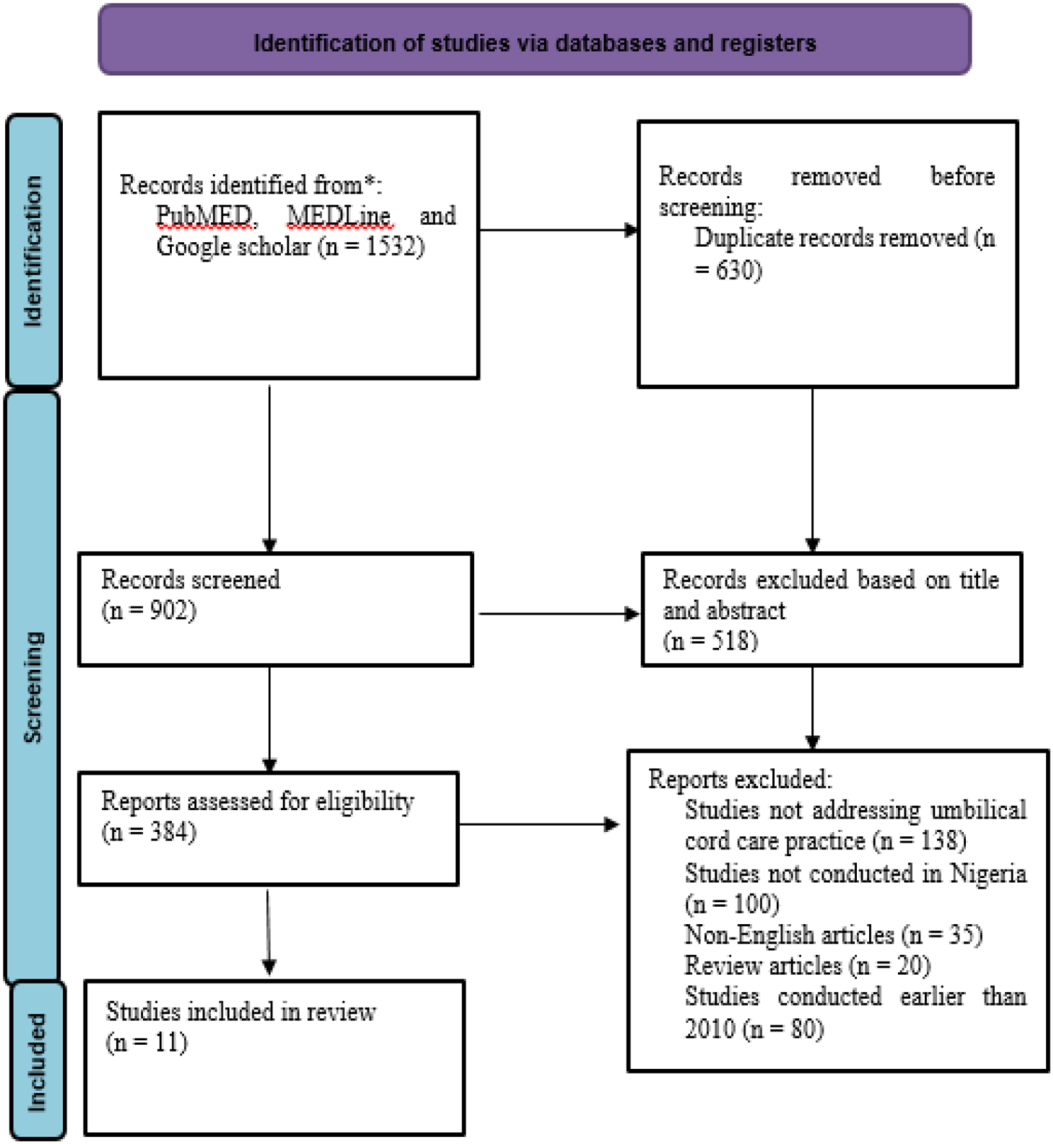
PRISMA Flow Diagram Showing the Study Selection Process [29, 35]

The study selection process commenced through the use of EndNote reference software for de-duplication of studies [28]. This process was performed by, DCI, PEA, ICD, VCM, and PCU. The studies were sorted based on their titles and abstracts against the eligibility criteria. Subsequently, full-text screening was performed to remove studies that did not match the inclusion criteria. The criteria for excluding studies were recorded in the PRISMA flow diagram (Fig. 1) [29].

### Data extraction

Microsoft Excel software was employed to extract relevant data from selected studies [30]. The following information was obtained from these studies: the study author and date, the study aim or title, the sample size and participant characteristics. Also other information extracted were the study design the methodology, the outcome of interest, and the study setting. These items were summarised in text and shown in a table by PEA, ACU, UGE, DCQ, PEA, CCO and CPA.

### Quality assessment

The Critical Appraisal Skills Programme (CASP) was performed by PEA, DCI, PEA, ICD, VCM, and PCU to assess the quality of this review. The Critical Appraisal Skills Programme (CASP) checklist is a widely used instrument developed to help individuals evaluate the quality and rigour of research studies, mainly in the disciplines of healthcare and social sciences [31]. It evaluates the quality of selected studies based on the following domains: research question or aim, study design, data collection, data analysis, result presentation, interpretation of findings, acknowledgement of limitations, relevance of findings, conclusion, ethical consideration, and conflicts of interest [31, 32]. The CASP checklist is a comprehensive and widely used set of criteria for evaluating the quality of research projects [31].

### Data synthesis

Data synthesis entails combining and evaluating results from different studies to make conclusions and generate new insights [33]. It is often used in research to summarise and make sense of huge amounts of data or information, particularly in systematic reviews, meta-analyses, and other evidence-based methodologies [33]. This review employed narrative synthesis because of its ability to meticulously review and interpret information from various sources, frequently applying a qualitative technique to construct a logical narrative that elucidates patterns, themes, and correlations in the data [34]. Narrative synthesis is often used when the data is too diverse or heterogeneous to undertake a quantitative analysis [34].

## Results

### Overview of the search process

We conducted a thorough literature search across the electronic databases, revealing a total of one thousand, five hundred and thirty-two (1532) studies. After the de-duplication and screening processes, eleven (11) studies were selected for this review. Figure 1 shows the PRISMA flow diagram illustrating the study selection process.

### Characteristics of included studies

The eleven selected studies covered in this review identified a total of 2540 study participants. Three of the studies were conducted in the South East geopolitical zone. Two of the included studies were carried out in northern Nigeria. One of the selected studies was conducted in the South Western geopolitical zone. Five out of the six studies were conducted in the Southern-Southern geopolitical zone. Eight studies were quantitative, while three employed qualitative approaches. Table 3 summarizes the characteristics of the listed studies.

**Table 3:** Characteristics of included studies

Author and Date of Publication	Aim/Title	Sample size and Participant characteristics	Study design	Practices/Measures	Study setting
Israel, et al., 2023	Knowledge and use of chorhexidine gel in umbilical cord care among postpartum women at Poly General Hospital, Enugu, Southeast Nigeria: a cross-sectional study	197 Postpartum women	Cross-sectional descriptive survey	From the study, A high percentage of the participant used chlorhexidine gel for umbilical cord care while a smaller percentage used it for only their last baby	Poly General Hospital Asata, Enugu State, Nigeria
Agu, et al., 2022	Umbilical Cord Care Knowledge and Practice: What is the Status of National Chlorhexidine Gel Scale-Up in Nnewi Nigeria?	193 Mothers	Cross-sectional study	From the study, Majority of respondents practiced the standard method of cord care using methylated spirit.	Nnewi.
Okpaleke, Ndikom, and Bulama,2019	To compare the incidence of umbilical cord infection between neonates receiving 7.1% chlorhexidine gel (CHG) and Methylated-spirit (MTS) in Ibadan	179 Newborn	Prospective-comparative study	The study exhibited a higher noncompliance rate among the chlorhexidine gel group than the Methylated-spirit. The participants made of the chlorhexidine gel while a higher participant made use of Methylated-spirit for umbilical cord care.	Ibadan
Mohammed, et al., 2020	Assessment of knowledge and cord care practices among pregnant women in selected PHCs in Jos metropolis, Plateau state	119 Pregnant women	Cross-sectional study	A significant number of the participant used methylated spirit in their last child delivery, a minute number used chlorhexidine gel while a similar low percentage used substances such as salt and vaseline.	Jos metropolis, Plateau state.
Ango, et al., 2021	Knowledge and practices of umbilical cord care among mothers attending antenatal care in the health facilities in Sokoto Metropolis, Nigeria	363 Pregnant women	Cross-sectional study.	The study revealed that the umblical cord are being cut using surgical blade and new razor blade. The materials used to tie the cord included cord clamp, sewing thread, and hair thread, the mothers also clean the base of the cord first before the surrounding. A higher percentage of mothers also practice proper hand washing before and after cleaning	Sokoto metropolis, Nigeria
Osuchukwu, Okoronkwo and Ezeruigbo, 2018	The Umbilical cord care and management outcome among mothers in Calabar South Local Government Area of Cross River State	451 Women of child bearing age	A cross sectional community-based study was conducted in	From the study, most of the respondents (49.8%) used methylated spirit in cleaning the cord, others used dettol (19.6%), saliva and salt 44(9.8%), herbal preparations (9.8%). Most of the respondents (69.8%) applied unhygienic substances at the base of the stump after cleaning the cord.	Calabar South Local Government Area of Cross River State, Nigeria.
Lawrence, et al., 2015	The umbilical cord care practices by traditional birth attendants in Yenagoa, Nigeria	31 Traditional birth attendant	A qualitative study design	From the study, only few trained TBAs saw the need to wash their hand before and after cord care. They often use razor blade to cut the cord while black thread and peg is commonly used to tie and clamp the umbilical cord respectively. In cases of emergency, they use a rope from sac bag to tie the cord. This study also reveal that substances mostly used by TBAs for cord care are methylated spirit, and local herbs.	Yenagoa Local Government Area, Bayelsa State, Nigeria
Asiegbu, et al., 2019	The determinants of cord care practices among mothers in Abakaliki, Ebonyi State, South East, Nigeria	273 Mothers	Cross-sectional study	Findings from this study showed that educated mothers utilized methylated spirit and chlorhexidine as a form cord care while those who had primary or no formal education made use of hot water, Vaseline, tooth paste and local herbs as a form of cord care.	Abakaliki, Ebonyi State, South East, Nigeria
Opara, Jaja and Okari, 2012	Newborn cord care practices amongst mothers in Port Harcourt, Nigeria	210 Mothers presenting with children 0- 6months old to the Teaching Hospital	Cross-sectional study	The study revealed that a very high percentage (95.3%) of mothers attending the clinic used methylated spirit to clean the cord, a smaller percentage (32.4%) applied potentially dangerous substances to the baby’s cord after cleaning with methylated spirit. Some of these substances included petroleum jelly, hot balms, tooth paste, herbs, occlusive dressings with methylated spirit soaked swabs and antibiotic ointments.	Paediatric Outpatient and Infant Welfare Clinics of the University of Port Harcourt, Nigeria.
AbhulimhenIyoha and Ibadin, 2012	Determinants of cord care practices among mothers in Benin City, Edo State, Nigeria	497 Mothers	Quantitative study	From the study, Most of the delivery units used thread and lower percentage used plastic cord clamp to secure haemostasis at the umbilical stump. Other materials used include suture materials, strips of cloth, bandage, plaster and rubber band. Also, majority of the participants practiced hand washing before and after cord care. The traditional practices of cord care in Benin City include the use of hot compress menthol-containing balm, herbs, native chalk, petroleum jelly, palm oil, toothpaste, salt, sand and saliva. Though the most common single agent for cord treatment was alcohol (methylated spirit).	University of Benin Teaching Hospital (UBTH), Benin City, Edo State, Nigeria
Duru, et al., 2023.	Sociocultural practices, beliefs, and myths surrounding newborn cord care in Bayelsa State, Nigeria	24 women and 3 traditional birth attendants	Qualitative study	From the study, the Traditional birth attendant usually cuts the infant’s cord with a razor blade and ties the stump with hair or sewing thread. The substances used for cord care included methylated spirit, “*African never-die”* leaf, and “*Close-Up”* toothpaste.	Bayelsa State, Nigeria

### Quality assessment/ critical appraisal

To evaluate the quality of the 11 selected studies, the CASP checklist was employed. According to the chart presented in Table 4, “no” implies that the criteria were not adequately addressed, “yes” indicates that the criteria were effectively addressed, and “not clear” suggests that there is insufficient evidence to draw a clear decision. “Yes” was awarded 1 point, “no” was given 0, and “while not clear” was given 0.5 points. Studies that scored 8– 11 were graded “high.” Studies with scores of 1–4 were classified as “low,” while those with scores of 5–7 were graded “medium.”

**Table 4:** Quality assessment of included studies

Study Author	Research Question or Aim	study design	data collection	data analysis	Result presentation	Interpretation of Findings	Acknowledgment of Limitations	Relevance of findings	Conclusion	Ethical consideration	Conflicts of Interest	Overall rating
Israel, et al. (2023)	Yes	Yes	Yes	Yes	Yes	Yes	Not clear	Yes	Yes	Yes	Yes	10.5/11 (High)
Agu, et al. (2022)	Yes	Yes	Yes	Yes	Yes	Yes	Yes	Yes	Yes	Yes	Yes	11/11 (High)
Okpaleke, Ndikom, and Bulama (2019)	Yes	Yes	Not clear	Yes	Yes	Yes	Not clear	Yes	Yes	Yes	Yes	10/11 (High)
Mohammed, et al. (2020)	Yes	Yes	Yes	Yes	Yes	Yes	Not clear	Yes	Yes	Yes	Yes	10.5 /11 (High)
Ango, et al. (2021)	Yes	Yes	Yes	Not clear	Yes	Yes	Yes	Yes	Yes	Yes	Not clear	10/11 (High)
Osuchukwu, Okoronkwo and Ezeruigbo, (2018)	Yes	Yes	Yes	Yes	Yes	Yes	Not clear	Yes	Yes	Not clear	Yes	10/11 (High)
Lawrence, et al. (2015)	Yes	Yes	Yes	Not clear	Not clear	Yes	Not clear	Yes	Yes	Not clear	Yes	9/11 (High)
Asiegbu, et al. (2019)	Yes	Yes	Yes	Yes	Yes	Yes	Yes	Yes	Yes	Not clear	Yes	10.5/11 (High)
Opara, Jaja and Okari (2012)	Yes	Yes	Yes	Yes	Yes	Yes	Not clear	Yes	Yes	Not clear	Yes	10/11 (High)
AbhulimhenIyoha and Ibadin (2012)	Yes	Yes	Yes	Yes	Yes	Yes	Not clear	Yes	Yes	Not clear	Yes	10/11 (High)
Duru, et al. (2023)	Yes	Yes	Yes	Yes	Yes	Yes	Not clear	Yes	Yes	Yes	Yes	10.5 /11 (High)

### Findings

The study highlighted seven key findings: cultural practices and beliefs; healthcare infrastructure and accessibility; traditional vs. modern approaches; barriers to adoption; impact of socioeconomic factors; prevalence of infections and complications; and effectiveness of interventions. Table 5 represents the key findings of the review.

**Table 5:** Key findings

Author/ Date	Cultural practices and beliefs	Healthcare Infrastructure and Accessibility	Traditional vs. Modern Approaches	Barriers to Adherence	Impact of Socioeconomic Factors	Prevalence of Infections and Complications	Effectiveness of Interventions
Israel, et al., 2023 [37]	Yes	Yes	Yes	Yes	Yes		Yes
Agu, et al., 2022. [38]		Yes	Yes		Yes		
Okpaleke, Ndikom, and Bulama, 2019 [39]						Yes	Yes
Mohammed, et al., 2020. [40]		Yes		Yes	Yes		Yes
Ango, et al., 2021. [41]	Yes				Yes		
Osuchukwu, Okoronkwo and Ezeruigbo, 2018. [42]						Yes	
Lawrence, et al., 2015. [43]	Yes		Yes				
Asiegbu, et al., 2019. [44]		Yes		Yes	Yes		
Opara, Jaja and Okari, 2012. [45]						Yes	Yes
AbhulimhenIyoha and Ibadin, 2012. [14]	Yes						
Duru, et al., 2023. [39]	Yes		Yes	Yes		Yes	Yes

#### Cultural practices and beliefs

Cultural Practices and Beliefs emerge prominently from this review, drawing insights from five relevant studies conducted in various regions [14, 36–39]. The cross-sectional study, which was carried out by Israel et al. [36] at Poly General Hospital in Enugu, demonstrated that different cultural backgrounds influence the application of different substances for umbilical cord care. The study found that people from various cultural backgrounds in Southeast Nigeria utilised different substances for umbilical cord care, highlighting the impact of cultural practices on newborn care [36]. This is supported by the findings of Ango et al. [37], who investigated mothers’ knowledge and habits about umbilical cord care in Sokoto Metropolis. According to the study, unsanitary cord cutting and the use of substances like sand mixed with saliva, herbal preparations, ashes, palm oil, and groundnut oil are common, demonstrating the impact of cultural beliefs on cord care practices [37]. Furthermore, Lawrence et al. [38] investigated the practices of traditional birth attendants (TBAs) in Yenagoa, focussing on the role of these traditional practitioners in cord care. TBAs employed local herbs and methylated spirits, demonstrating how cultural practices influence the selection of products for umbilical cord care [38]. Duru et al. [39] also did a qualitative study in Bayelsa State to investigate sociocultural ideas and practices about cord care. The findings highlighted the importance of cultural traditions in TBA delivery and the use of substances such as methylated spirit, “African never-die” leaves, and “Close-Up” toothpaste [39]. Finally, Abhulimhen Iyoha and Ibadin [14] observed harmful cord care practices in Benin City, with traditional practices such as hot compress, menthol-containing balm, herbs, and native chalk being common, emphasising the persistence of cultural beliefs influencing cord care practices. Overall, these studies highlight the crucial importance of cultural practices and beliefs in defining Nigerian umbilical cord care practices [14, 36–39].

#### Healthcare infrastructure and accessibility

Four relevant studies have shown that the issue of “healthcare infrastructure and accessibility” is important in the understanding of this review [36, 40–42]. For example, Agu et al.‘s [40] study emphasised the importance of healthcare infrastructure, revealing that the majority of mothers who participated in the study had access to health facilities with skilled providers for delivery, highlighting the role of accessible healthcare services in influencing cord care practices [40]. Furthermore, Mohammed et al. [41] evaluated cord care practices among pregnant women in selected primary health care facilities in Jos metropolis, Plateau State, emphasising the importance of healthcare infrastructure, including the availability of health education during antenatal care, in influencing cord care knowledge and practices among mothers [41]. The study conducted at Poly General Hospital in Enugu by Israel et al. [36] provides insights into healthcare accessibility, reporting on a cross-sectional survey of postpartum women and the need for healthcare providers to continue providing information on optimal umbilical cord care to improve knowledge and practices. Furthermore, Asiegbu et al. [42] in Abakaliki investigated the determinants of cord care practices and found that maternal education, occupation, and parity influence the care given to the umbilical cord after birth, emphasising the interconnectedness of healthcare infrastructure and individual characteristics. The importance of the healthcare system in maternal and neonatal care studies was reflected in the consideration of healthcare infrastructure and accessibility in shaping umbilical cord care practices in Nigeria [36, 40–42].

#### Traditional vs. modern approaches

Traditional or home remedies are frequently contrasted with modern medical interventions in umbilical cord care, reflecting a divergence in approaches influenced by cultural beliefs and healthcare practices, as evidenced by multiple studies [36, 38–41]. Traditional methods, as observed in studies such as those conducted by Israel et al. (2023) and Lawrence et al. (2015), frequently involve the application of culturally specific substances to the umbilical stump, such as herbal preparations, saliva, or local herbs, demonstrating deeply rooted practices passed down through generations [36, 38]. Modern medical interventions, on the other hand, emphasised evidence-based practices supported by healthcare providers, such as the use of antiseptic solutions like chlorhexidine gel and health education during antenatal care to promote optimal cord care, as shown in other studies [40, 41]. Duru et al. (2018) conducted a community-based study in Calabar South Local Government Area, discovering a variety of traditional substances used, including methylated spirit, dettol, saliva, salt, herbal preparations, and unsanitary materials at the stump’s base, highlighting the coexistence of traditional and modern practices [39]. According to Duru et al. (2018), the difference is that modern interventions aim to reduce such risks through the use of medically approved drugs, whereas traditional techniques may be associated with unsanitary practices and an increased risk of infection [39]. The comparison highlights the importance of a nuanced understanding of cultural contexts as well as the incorporation of evidence-based medical interventions to ensure the best outcomes for newborns in a variety of healthcare settings [36, 38–41].

#### Barriers to adherence

Barriers to Adherence was prominent in this review which drew insights from five included studies [38–42]. A study in Nnewi identified barriers to the adoption of recommended cord care practices, specifically the almost non-existent awareness and use of chlorhexidine gel despite its national scale-up, indicating potential knowledge gaps and communication challenges as barriers to adherence [40]. Another study investigated the sociocultural behaviors, beliefs, and myths surrounding infant cord care in Bayelsa State, uncovering deeply embedded cultural elements as impediments to adherence [39]. The study found that sociocultural beliefs influenced preferences for TBAs and the use of substances such as methylated spirit, “African never-die” leaf, and “Close-Up” toothpaste, posing challenges to the acceptance of recommended medical interventions [39]. The activities of TBAs were explored in Yenagoa, shedding light on how cultural customs influenced cord care decisions and highlighting potential resistance to modern medical guidelines as impediments to adherence [38]. Furthermore, the predictors of cord care practices in Abakaliki were found highlighting maternal education, occupation, and parity as potential hurdles, implying that individual traits may hamper adherence to standardized cord care methods [42]. Furthermore, a study in Jos discovered that, despite having a strong understanding of cord care standards, a significant number of mothers employed a combination of septic and aseptic approaches, highlighting a potential barrier to adherence to recommended aseptic practices [41]. These studies, taken together, highlight the presence of barriers, such as traditional beliefs, cultural practices, and a lack of awareness, that may impede adherence to recommended umbilical cord care practices in various regions of Nigeria [38–42].

#### Impact of socioeconomic factors

Five studies provided insights on the “Impact of Socioeconomic Factors,” revealing how socioeconomic conditions influence mothers’ choices and behaviours [36, 37, 40–42]. Agu et al. [40] conducted a study that highlighted the importance of the examined mothers’ average age and socioeconomic level in moulding cord care habits, demonstrating the interconnection of socioeconomic determinants with maternal practices [40]. Asiegbu et al. [42] investigated the factors that influence cord care practices, discovering that maternal education, occupation, and parity were related to the type of care given to the umbilical cord after birth, highlighting the importance of socioeconomic factors in decision-making. In Jos, Mohammed et al. [41] discovered that respondents with university education were more likely to employ aseptic cord care procedures, emphasising the importance of educational attainment, a socioeconomic indicator, on cord care choices. Furthermore, Israel et al. [36] investigated chlorhexidine gel knowledge and use, identifying socioeconomic factors such as educational status as significantly associated with its use and emphasising the role of socioeconomic indicators in determining adherence to evidence-based practices. Ango et al. [37] investigated the knowledge and practices of umbilical cord care among antenatal care mothers and identified factors such as educational level and occupation, indicating that socioeconomic factors play a role in shaping cord care practices in health facilities. These studies show that socioeconomic characteristics, notably maternal education, have a significant impact on umbilical cord care practices in Nigeria [36, 37, 40–42].

#### Prevalence of infections and complications

Five included studies [39, 43–45] have shed light on the prevalence of infections and complications. Osuchukwu et al. [44] conducted a community-based study and discovered that the majority of respondents applied unsanitary substances to the umbilical cord, resulting in a high prevalence of umbilical cord infections, highlighting the link between suboptimal cord care practices and the prevalence of complications. In addition, Duru et al. [39] conducted a community-based study and discovered that the majority of respondents applied harmful and contaminated materials to the umbilical cord, resulting in a high prevalence of infected cords, highlighting the potential consequences of suboptimal cord care practices on neonatal health. Okpaleke et al. [43] conducted a comparative study and discovered that the incidence of umbilical cord infection did not differ significantly between neonates treated with 7.1% chlorhexidine gel and those treated with methylated spirit, highlighting the importance of investigating the efficacy and potential complications associated with different cord care interventions. Furthermore, Opara et al. [45] investigated newborn cord care procedures in Port Harcourt and discovered that 2.9% of mothers reported cord issues, underscoring the potential complications connected with cord care practices even in a mostly urban area. These studies, taken together, highlight the importance of investigating the prevalence of infections and complications associated with various cord care practices in Nigeria, as well as the need for evidence-based interventions to reduce morbidity and improve neonatal outcomes [39, 43–45].

#### Effectiveness of interventions

Insights on the “effectiveness of interventions” in umbilical cord care practices were provided in five of the relevant studies [36, 39, 41, 43, 45]. Okpaleke et al. [43] conducted a prospective-comparative study, comparing the incidence of umbilical cord infection between neonates receiving 7.1% chlorhexidine gel and methylated spirit, and found no statistically significant difference in infection rates between the two groups, implying that both interventions were equally effective in preventing cord infections [43]. In contrast, Israel et al. [36] investigated the knowledge and use of chlorhexidine gel in Enugu, discovering suboptimal utilisation and low awareness, indicating potential limitations in the effectiveness of health education interventions aimed at increasing chlorhexidine use in the studied population (Israel et al., 2023). Similarly, Mohammed et al. [41] assessed knowledge and cord care practices among pregnant women in Jos, finding that despite good knowledge of cord care practices, a significant number of mothers used a combination of septic and aseptic methods, indicating a potential gap in the effectiveness of health education interventions [41]. Opara et al. [45] investigated infant cord care practices in Port Harcourt, finding 2.9% of mothers had cord issues, indicating a potential gap in the effectiveness of current cord care methods in an urban setting and underscoring the need for enhanced interventions. Duru et al. [39] conducted a community-based study in Calabar South and discovered a high incidence (75.1%) of infected umbilical cords, highlighting potential limitations in the effectiveness of current cord care practices in the community. These studies underscore the need for greater investigation and evaluation of the effectiveness of existing interventions in Nigerian umbilical cord care practices, pointing to the need for evidence-based guidelines to improve the overall efficacy of newborn care measures.

### Summary of findings/ themes

This review revealed a complex environment with a variety of influences. According to studies like those by Lawrence et al. [38] and Duru et al. [39] which emphasized the ongoing practice of traditional treatments involving the use of local herbs, saliva, and unsanitary materials, cultural practices and beliefs are significant. Traditional and modern techniques coexist, stressing the importance of the cultural environment in determining cord care practices [39]. According to Duru et al. [39], the disparity between traditional and modern approaches emphasized the necessity of acknowledging cultural influences and combining evidence-based strategies for effective umbilical cord care. Studies by Agu et al. [40] and Mohammed et al. [41] shed light on discrepancies in knowledge and practices, pointing to potential hurdles in healthcare delivery and the need for focused interventions. Asiegbu et al. [42], Agu et al. [40], and Mohammed et al. [41] emphasized the importance of socioeconomic determinants, particularly maternal education, exposing the connection between educational attainment and adherence to recommended cord care methods. Adherence barriers are emphasized in research by Israel et al. [36], Mohammed et al. [41] and Duru et al. [39] which indicated gaps in knowledge, awareness, and execution of evidence-based cord care procedures. Finally, research such as Okpaleke et al. [43] and Duru et al. [39] investigated the prevalence of infections and complications, highlighting differences in outcomes and emphasizing the need for complete understanding to improve newborn health outcomes in Nigeria. Okpaleke et al. [43] examine the effectiveness of interventions, particularly the use of chlorhexidine gel, underscoring the need for evidence-based solutions to enhance cord care practices. These findings illustrate a complex landscape of umbilical cord care practices in Nigeria, highlighting the significance of culturally appropriate, accessible, and effective treatments to maximize newborn care.

### Discussion of findings

Diverse outcomes appeared throughout the selected studies in the review, contributing to a thorough grasp of the complexities involved. Israel et al.‘s [36] study in Enugu found a serious knowledge gap about chlorhexidine gel use among postpartum mothers, with more than half demonstrating insufficient knowledge. Similarly, Agu et al. [40] found little understanding and use of chlorhexidine gel in Nnewi, underlining the need for improved education and awareness initiatives. Okpaleke et al.‘s [43] analysis in Ibadan revealed a comparable lack of understanding and use of chlorhexidine gel, emphasizing the ongoing difficulties in implementing evidence-based procedures. In contrast to these findings, Mohammed et al. [41] in Jos discovered differences in cord care habits, with a large number of women preferring methylated spirit. Ango et al.‘s [37] study in Sokoto indicated a significant gap in cord care behaviors, with a significant proportion of women demonstrating inadequate practices while having good knowledge. The study conducted by Osuchukwu et al. [44] in Calabar South shed light on various cord care techniques, revealing that a significant percentage of moms applied toxic substances to the umbilical cord, posing possible hazards. Similarly, Lawrence et al. [38] discovered habits such as utilizing a razor blade for cord cutting in their research of TBAs in Yenagoa, highlighting the importance of recognizing and adapting ancient traditions into modern healthcare approaches. Furthermore, Abhulimhen Iyoha and Ibadin [14] discovered a prevalence of dangerous cord care practices in Benin City, with the majority of mothers using thread and plastic cord clamps, indicating a vital need for interventions to prevent such potentially risky behaviors. The research by Asiegbu et al. [42] in Abakaliki revealed detrimental habits, notably among women with lower educational levels, underlining the relevance of education in determining cord care decisions. The findings of Opara et al.‘s [45] analysis in Port Harcourt highlighted the ubiquity of potentially hazardous compounds used for cord care, health outcomes in Nigeria.

#### Future practices

The implications of this review have far-reaching significance for shaping the country’s future healthcare practices. The discovered knowledge gaps, notably with regard to the underutilization of chlorhexidine gel, indicate a critical need for specialized training programs in future healthcare practices. Future practitioners should prioritize including evidence-based information on optimal cord care in their interactions with expecting mothers, emphasizing the benefits and proper administration of chlorhexidine gel. Furthermore, the prevalence of harmful cord care practices highlighted the significance of future regulations that aggressively discourage the use of potentially hazardous substances, promote the adoption of safe alternatives, and ensure the availability of suggested items. Because traditional birth attendants will continue to be used, future healthcare approaches must welcome and engage with them. Training programs should be established to integrate traditional methods with evidence-based concepts, promoting a harmonious blend of traditional and modern cord care treatments. According to the review’s findings on sociocultural barriers, future healthcare practices must be culturally sensitive and community-focused. Integrating cultural competence training into healthcare education and establishing community-based projects are key steps toward narrowing the gap between traditional and modern healthcare practices. Future healthcare practices should aggressively engage communities in open-cord care debates in order to overcome deeply ingrained views and habits. To address imbalances created by socioeconomic factors, future healthcare operations must employ a holistic plan. Strategies should prioritize increasing access to healthcare resources, supporting maternal and newborn care, and addressing broader societal issues. Future healthcare practices must be designed with the purpose of reducing health inequities and delivering high-quality, evidence-based care to all mothers and newborns. In conclusion, the findings of the systematic review direct future healthcare practices in Nigeria toward a more informed, culturally sensitive, and equitable approach to umbilical cord care, with the ultimate goal of improving mother and newborn health outcomes across the country.

### Strengths and limitations of the systematic review

#### Strengths

This systematic review has various strengths that contribute to the robustness and dependability of its findings. First and foremost, this review took a comprehensive approach by including a varied variety of research completed in various regions of Nigeria, providing a comprehensive look at cord care methods across the country. The inclusion of both qualitative and quantitative studies added to the review’s richness, providing for a more nuanced understanding of the cultural, societal, and healthcare aspects that influence cord care. The methodical search and selection procedure, as evidenced by the precise inclusion criteria and clear methodology, contributed to the reduction of bias and the inclusion of relevant and high-quality studies. Furthermore, narrative synthesis of findings were categorized based on key findings, such as cultural practices, healthcare infrastructure, and socioeconomic considerations, provide a systematic and clear presentation of the various aspects influencing umbilical cord care. These factors contributed to the systematic review’s conclusions being more reliable and applicable, making it a valuable resource for directing future research directions and healthcare policies linked to umbilical cord care practices in Nigeria.

#### Limitations

This review, notwithstanding its strengths, is not without limitations. One significant limitation is the possibility of publication bias, as the review may have mistakenly eliminated unpublished research or those published in languages other than English. This could result in an insufficient representation of the available evidence, thus impacting the findings’ generalizability. Furthermore, some studies’ dependence on self-reported data may include recollection bias and social desirability bias, reducing the accuracy of reported cord care habits. The differences in study designs and methodology among the included studies may make it difficult to synthesize the results and draw broad conclusions. Furthermore, the review’s omission of papers conducted prior to 2010 may leave out crucial historical context and trends in Nigerian cord care practices. Finally, the variability in sample sizes and participant demographics among researchers may restrict the comparability of findings. Recognizing these limitations is critical for cautiously interpreting the review’s findings, and it highlights the need for future research to fill these gaps for a more thorough understanding of umbilical cord care practices in Nigeria.

### Conclusion

In conclusion, this systematic review provided a thorough examination of the existing literature, shining light on many factors influencing maternal and neonatal health. This review shed light on the various cultural practices, healthcare infrastructure issues, socioeconomic considerations, and traditional versus modern techniques that shape umbilical cord care in Nigeria. While identifying knowledge gaps and potential intervention areas, the research underlined the importance of evidence-based educational activities, community participation, and partnerships with traditional birth attendants. The findings and synthesis emphasized the significance of future healthcare practices that are culturally sensitive, equitable, and socially inclusive. The study has significant merit, offering valuable insights that could positively impact the pediatric community. By influencing healthcare policies, guiding future research, and improving umbilical cord care practices, the findings could enhance maternal and newborn outcomes in Nigeria. If the proposed recommendations—such as addressing knowledge gaps, promoting safe practices, and integrating culturally sensitive approaches—are effectively implemented, the benefits for both maternal and neonatal health could be substantial.

## Data Availability

The datasets used and/ or supplementary files analysed during this current study are available from the corresponding author upon reasonable request.
